# Analysis of thyroid function and related factors in narcolepsy patients

**DOI:** 10.1038/s41598-023-45321-x

**Published:** 2023-10-28

**Authors:** Hongli Wang, Mingrui Jia

**Affiliations:** Department of Pain Management, The Second Hospital of Shandong University, Jinan, 250033 Shandong China

**Keywords:** Neuroscience, Diseases

## Abstract

The loss of hypocretin is thought to be the main pathophysiological mechanism of narcolepsy. There is strong evidence that hypocretin is related to the regulation of endocrine functions and depression. To explore thyroid hormone levels in narcolepsy patients was our aim. In addition, further is to analyze the relationship between thyroid hormone levels and sleep quality, anxiety, and depression in narcolepsy patients. There are 40 patients with narcolepsy and 40 healthy controls (HCs) were conducted. Blood samples were explored for thyroid function. Correlation analysis between thyroid hormones and clinical characteristics of narcolepsy was performed using Pearson or Spearman. Narcolepsy patients had significantly lower free thyroxine (FT_4_) levels in comparison to controls (p < 0.001). No subject was diagnosed with primary hypothyroidism. There were 4 (10%) subjects with subclinical hypothyroidism. The serum FT4 levels were positively correlated with HAMA_14_ score (r = − 0.343, p = 0.030) by Pearson correlation analysis. The serum TSH levels and HAMD_24_ score (r = − 0.807 p ˂0.001), and ESS score (r = − 0.317, p = 0.046) both showed a negative correction. Hypocretin deficiency may be associated with the regulation of thyroid hormones in narcolepsy patients. The serum thyroid hormones may affect the severity and neuropsychological functions of narcolepsy patients.

## Introduction

Nacolepsy type 1 (NT1) is associated with cataplexy attacks, orexin deficiency, and excessive daytime sleepiness (EDS)^1,2^. It is estimated that approximately affects 1/2000 individuals^3^. The destruction of hypocretin neurons were thought to be the autoimmune-mediated, which was the main pathophysiological mechanism of NT1^4–6^.Besides, more than 98% of NT1 with cataplexy patients are positive for HLA-DQB1*0602^6–8^. Additionally, more evidence has demonstrated that environmental factors and genetic also involved in the pathogenic mechanism of NT1^9^.

Recently, the association between NT1 and thyroid disease is attractive, which supports the hypothesis of a causal autoimmune disorder or co-existing ones^10^. A previous study has found that NT1 patients may have concomitant thyrotropin deficiency^11^. However, the results were conducted from only seven NT1 patients, which is limited to relatively small numbers of subjects to draw firm conclusions. Interestingly, a case report found significant improvement in EDS symptoms with levothyroxine treatment in a euthyroid patient with NT1^12^. There is strong evidence that hypocretin is related to the regulation of endocrine functions and depression^13^. Besides, a previous study showed that the hypothalamic-pituitary-thyroid (HPT) axis can be modulated by hypocretin^14^. In addition, electrical stimulation of the lateral hypothalamic area (LHA) has been demonstrated to induce morphological changes in the thyroid gland^15^. As accumulated pieces of evidence suggested that neurophysiological and behavioral processes of mood disorders can be significantly affected by hypocretin^13^. Thyroid hormone is involved in regulating psychological function and emotion^16^. The relationship between the HTA axis and mood disorder has been increasingly studied^17^. Growing evidence have indicated that NT1 patients have an increased prevalence of psychiatric comorbidities^18^. Our aim was to assess differences in serum thyroid hormone levels between patients with NT1 and healthy controls (HCs) in this case–control study.

## Materials and methods

### Research subjects

This case–control study was conducted from 2019 to 2022. There are 40 NT1 patients and 40 healthy individuals during the same period. The International Classification of Sleep Disorders (ICSD-3) diagnostic criteria was used to confirm NT1 diagnosis^19^. The healthy controls selected from the general population via advertisement and local association networks, no objective sleepiness on the MSLT (mean sleep latency > 8 min), no medication use, and no other significant psychiatric, neurologic, or medical disorders.

The study was approved by the Ethics Committee of The Second Hospital of Shandong University, and all participants signed written informed consent prior to participation. This study was conducted in accordance with the Declaration of Helsinki.

### Exclusion criteria

Exclusion criteria were as follows: (1) medical history of central nervous system demyelinating disease, central nervous system infection, and intracranial tumors; (2) patients with a history of metabolic illness(e.g. autoimmune liver disease, viral hepatitis, diabetes, and hypertension) were excluded; (3) patients with a history of ^131^I treatment for hyperthyroidism, neck radiotherapy, thyroidectomy were excluded; (4) Exclusion of participants with comorbid other forms of sleep disorders (e.g. OSA, RLS); (5) Comorbid psychiatric disorders were excluded^20^.

### Research methods

#### Collecting general information

To assess the subjects’ EDS symptoms, the Epworth sleepiness scale (ESS) was used by professional physicians^21^.

#### Laboratory assessment

After an overnight fast of 8 h, venous blood samples were collected from all subjects and serum free thyroxine (FT_4_), free triiodothyronine (FT3) and thyroid stimulating hormone (TSH) levels were assessed according to standard operating procedures, the Architect i2000SR automated immunoassay system was used to measure FT_4_, FT3 and TSH^20^. We used the ^125^I radioimmunoassay kit (Phoenix Pharmaceuticals, Belmont, CA, USA) to muesure the cerebrospinal fluid (CSF) hypocretin-1 levels.

#### Clinical and neuropsychological assessment

To measure the sleep quality, the Pittsburgh sleep quality index (PSQI) was used^20,22^. To measue the anxiety, the 14-item Hamilton Anxiety Scale (HAMA_14_) was used^20^. While, to measure the depression symptoms of narcoelpsy patients, the 24-item Hamilton depression rating scale (HAMD_24_) were used^20,23^.

### Statistical analysis

All analyses were performed using SPSS 24.0 computer software^20^. The mean ± standard deviation (SD) was uesed to reprent the continuous variables. The interquartile range (M, P_25_, P_75_) was used to present non-normally distributed data, which was tested by the student’s *t* test. Mann–Whitney *U* test was used to test the normality of the data. To compare the groups, we used the Chi-square test or Fisher’s exact test. Categorical data were expressed in amount (%)^20^. Spearman or Pearson for correlation analysis^20^. A P value of less than 0.05 was considered statistically significant.

### Ethics statement

The study was approved by the Ethics Committee of The Second Hospital of Shandong University (No.2021141), and all participants signed written informed consent prior to participation. This study was conducted in accordance with the Declaration of Helsinki..

## Results

### Demographics and clinical characteristics

**Figure 1:**
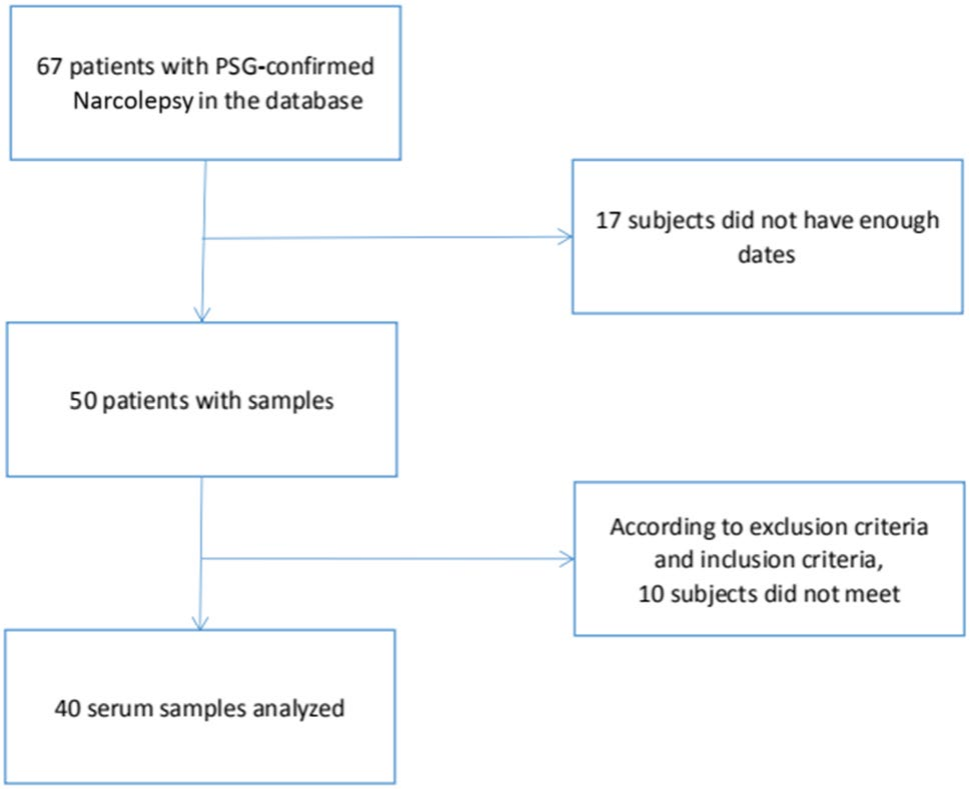
Flow chart of patient inclusion.

A total of 40 patients with NT1 were included in the study according to the inclusion and exclusion criteria (Fig. 1). Biochemical characteristics and demographic data characteristics of the participants are presented in Table 1.

**Table 1:** Demographic data and results of biochemical analyses.

	Narcolepsy (n = 40)	Control (n = 40)	*P* value
Gender (male/female)	28/12	23/17	0.245^a^
Age (years)	23.50 ± 10.89	26.20 ± 13.66	0.331^b^
BMI (kg/m^2^)	26.66 ± 3.73	25.28 ± 5.17	0.178^b^
SBP (mmHg)	124.92 ± 11.07	125.73 ± 11.19	0.749^b^
DBP (mmHg)	79.26 ± 8.75	80.02 ± 9.70	0.717^b^
Alcohol [n (%)]	5(10.0%)	6(12.5%)	0.745^a^
Smoking [n (%)]	5(10.0%)	6(12.5%)	0.745^a^
PSG			
AHI (event/h)	2.00 ± 1.58	2.40 ± 1.14	0.660^b^
PLMI (event/h)	1.85 ± 0.07	1.40 ± 0.14	0.092^b^
Neurological features			
PSQI	13.95 ± 3.23		
HAMA_14_	11.85 ± 3.97		
HAMD24	16.98 ± 4.71		
ESS total	21 (20.00, 21.00)		
Hypocretin-1 (pg/ml)	10.00 (10.00, 19.68)		
Duration of narcolepsy (year)	4.77 (2.00, 7.00)		
Age at symptom onset (years)	18.61 (10.25, 23.50)		

Data are presented as mean ± standard deviation, or median (interquartile range) as appropriate. The differences were considered significant if p-value < 0.05.

*BMI* body mass index defined as weight in kilograms divided by the square of height in meters, *SBP* systolic blood pressure, *DBP* diastolic blood pressure, *PSG* polysomnography, *AHI* apnea hypopnea index, *PLMI* rapid eye movement sleep latency, *ESS* Epworth sleepiness scale, *PSQI* Pittsburgh sleep quality index (PSQI) scale, *HAMD*_24_ 24-item Hamilton depression rating scale, *HAMA*_14_, 14-item Hamilton anxiety scale.

^a^χ^2^-test, ^b^*t* test.

### Comparison of thyroid hormones of NT1 patients and healthy subjects

The levels of FT4 in serum (*p* < 0.001) were significantly lower in patients with NT1 compared to the HC group. However, no differences were found between TSH (p = 0.906) or FT3 (p = 0.274) levels (Table 2).

**Table 2:** Serum levels of TSH, FT3 and FT4 in the Narcolepsy and HC groups.

	Narcoleps (n = 40)	Control (n = 40)	*P* value
FT3 (pmol/L)	5.05 ± 0.64	5.23 ± 0.78	0.274^b^
FT4 (pmol/L)	13.91 ± 1.62	15.36 ± 1.51	** < 0.001**^**b**^
TSH (uIU/mL)	2.57 ± 1.16	2.54 ± 1.15	0.906^b^

Data are presented as mean ± standard deviation, or median (interquartile range) as appropriate. The differences were considered significant if p-value < 0.05.

*TSH* thyroid stimulating hormone, *FT3* free triiodothyronine, *FT4* free thyroxine.

^a^χ^2^-tests, ^b^*t*-test, ^c^the Mann–Whitney *U*-test.

Significant values are in bold.

### The number of subjects in NT1 patients categorized by thyroid hormones

Table 3 shows the number of subjects in the NT1 group classified by FT4, FT3 and TSH levels. 4 (10%) patients had higher than normal TSH levels. 0 (0%) and 4 (10%) subjects had lower than normal FT3 and FT4 levels. No patient was diagnosed with primary hypothyroidism, compared to 4 (10%) subjects who were diagnosed with subclinical hypothyroidism. No hyperthyroidism was found in our study.

**Table 3:** Number of subjects categorized by TSH and FT3/FT4 levels.

		TSH			Total
		Low	Normal	High	
FT3	Low	0	0	0	0
	Normal	0	36	4	40
	High	0	0	0	0
FT4	Low	0	6	0^a^	4
	Normal	0	30	4^b^	36
	High	0	0	0	0
Total		0	36	4	40

Normal range of TSH, FT3, and FT4 were 3.1–6.8 pmol/L, 12.0–22.0 pmol/L, and 0.27–4.2 pmol/L, respectively.

*TSH* thyroid stimulating hormone, *FT3* free triiodothyronine, *FT4* free thyroxine.

^a^Primary hypothyroidism, ^b^Subclinical hypothyroidism.

### Correlation analysis of thyroid hormones and clinical characteristics of NT1

A statistically significant negative correlation was found between serum TSH levels and HAMD_24_ score (r =  − 0.807, *P*-value ˂0.001), and ESS score (r =  − 0.317,* P*-value = 0.046). The serum FT_4_ levels was positively correlated with HAMA_14_ score(r =  − 0.343, *P*-value = 0.030) by Pearson correlation analysis (Table 4).

**Table 4:** Correlation between thyroid hormones and clinical characteristics of Narcolepsy.

Item	TSH		FT3		FT4	
	r value	p value	r value	p value	r value	p value
PSQI score	0.174	0.282	0.166	0.305	0.064	0.674
HAMD_24_ score	− 0.807	** < 0.001**	0.016	0.922	− 0.157	0.332
HAMA_14_ score	− 0.064	0.697	0.087	0.594	− 0.343	**0.030**
ESS score	− 0.317	**0.046**	0.266	0.097	0.133	0.415
Hypocretin-1	− 0.118	0.469	− 0.205	0.203	− 0.208	0.198

*TSH* thyroid stimulating hormone, *FT3* free triiodothyronine, *FT4* free thyroxin, *ESS* epworth sleepiness scale, *PSQI* Pittsburgh sleep quality index (PSQI) scale, *HAMD*_24_ 24-item Hamilton depression rating scale, *HAMA*_14_ 14-item Hamilton anxiety scale.

Significant values are in bold.

## Discussion

### NT1 and thyroid function

Kok et al.^11^ reported that the serum levels of TSH were lower in patients with NT1. Szakacs et al.^24^ considered that the median TSH was decreased in NT1 patients with cataplexy. Our results also suggested that the FT_4_ was significantly decreased in NT1 patients. However, Chabas et al.^25^ found no different in serum TSH and FT_4_ levels. In addition, there was no difference in FT_3_ and TSH levels between NT1 patients and the control group. Moreover, in an autopsy study, the thyrotropin releasing hormone-expressing neurons were unaffected in paraventricular nucleus (PVN) of patients with NT1^26^. Thyroid hormone changes in patients with NT1 remain unknown due to the small number of studies and conflicting results. CG-3703, a thyroid-releasing hormone (TRH) analog can increase wakefulness, suppressed both REM sleep, slow-wave sleep, and significantly reduce cataplexy in canine NT1^27,28^. However, there were four (10.0%) subjects with subclinical hypothyroidism in our study. Hypocretin can regulate the HPT axis, which could provide insights into the understanding of the broad symptom spectrum of NT1. Martinez-Orozco et al.^29^ found an increased prevalence of autoimmune thyroid disease in NT1 patients, which may provide a valuable starting point for basic and applied research into the disease. However, autoimmune thyroid disease was not seen in our present study.

### Orexin system and HPT axis

Several animal experiments have already demonstrated that the hypocretin system can be able to modulate the HPT axis in rodents. Kaufman et al.^30^ found that the levels of thyroid hormone were lower in rats with LHA lesions. Besides, Suzuki et al.^31^ found that the administration of TRH in the LHA can significantly enhance the anorexia effect. In addition, studies have demonstrated that peripheral administration of hypocretin-1 can inhibit the TRH release from the hypothalamus in rats^32^. Different mechanisms for the causal relationship between thyroid function and NT1 were suggested. The PVN can receive hypocretin input signals^33^, which is also the central point of the regulation of the HPT axis^34^. Hypophysiotropic neurons of the PVN of the hypothalamus expressing TRH project to the portal system, via which it reaches the thyrotropin-producing cells of the anterior pituitary^35^. Thyroid hormone secretion is suppressed during starvation, while preprohypocretin mRNA is upregulated in the LHA^36^. We hypothesized that hypocretin neural circuits can modulate the HPT axis. Despite the evolving understanding of the hypocretin system, its role on the HPT axis in NT1 seems to remain unclear.

### Thyroid dysfunction and depression

The neuroendocrine dysfunction theory of the pathogenesis of depression mainly includes abnormalities of the hypothalamic–pituitary–adrenal axis and the HPA axis^10^. The relationship between thyroid function and depression has been of great interest to scholars, and it has been suggested that there are alterations in the function of the hypothalamic pituitary thyroid (HPT) axis in patients with depression, and it has been reported that disturbances in the level of thyroid hormones not only affects the patient's emotional response, but also serves as a marker of sensitivity in some patients with depression^15^. Studies have indicated that FT3 and FT4 may be the hallmark markers for predicting the degree of depression, and thyroid-related tests may be considered as routine examinations for adolescents with depression^16^. Hypothyroidism can induce depression and inattention, while mood disorders also be able to modulate the function of HPT axis^16^. Some researchers have proposed that thyroid hormones have an antidepressant effect in depression^37^. The serum TSH level of patients with NT1 was linked with the more severe subjective sleepiness symptoms, and the worse depression. In addition, we found that the serum FT_4_ levels was positively correlated with HAMA_14_ score. Hypocretin deficiency can induce the cholinergic monoaminergic imbalance, which may be involved in the pathophysiological process of depression^18^. However, although previous studies have found that thyroid hormones are involved in the pathogenesis of depression, the exact mechanism of the HPT axis in depression remains unclear.

### Limitations and recommendations

Firstly, subjects should be followed up periodically to assess their clinical characteristics as well as thyroid function, further illustrating the changes in thyroid hormones in patients with NT1. In addition, our results remain weak due to the sample size; our study subjects represent only NT1 patients with cataplexy.

## Conclusion

Hypocretin deficiency may be associated with the regulation of thyroid hormones in NT1 patients. The serum thyroid hormones may affect the severity and neuropsychological functions of NT1 patients. The exact relationship between thyroid hormones and NT1 should be validated in multi-center, and large-sample clinical studies in the future.

## Data Availability

The data that support the findings of this study are available on request from the corresponding author. The data are not publicly available due to privacy or ethical restrictions.
